# Age and CD161 Expression Contribute to Inter-Individual Variation in Interleukin-23 Response in CD8+ Memory Human T Cells

**DOI:** 10.1371/journal.pone.0057746

**Published:** 2013-03-01

**Authors:** Hui Shen, Wei Zhang, Clara Abraham, Judy H. Cho

**Affiliations:** 1 Department of Medicine and Genetics, Yale University School of Medicine, New Haven, Connecticut, United States of America; 2 Department of Medicine, Yale University School of Medicine, New Haven, Connecticut, United States of America; UTHealth Medical School, United States of America

## Abstract

The interleukin-23 (IL-23) pathway plays a critical role in the pathogenesis of multiple chronic inflammatory disorders, however, inter-individual variability in IL-23-induced signal transduction in circulating human lymphocytes has not been well-defined. In this study, we observed marked, reproducible inter-individual differences in IL-23 responsiveness (measured by STAT3 phosphorylation) in peripheral blood CD8+CD45RO+ memory T and CD3+CD56+ NKT cells. Age, but not gender, was a significant (Pearson’s correlation coefficient, r = −0.37, p = 0.001) source of variability observed in CD8+CD45RO+ memory T cells, with IL-23 responsiveness gradually decreasing with increasing age. Relative to cells from individuals demonstrating low responsiveness to IL-23 stimulation, CD8+CD45RO+ memory T cells from individuals demonstrating high responsiveness to IL-23 stimulation showed increased gene expression for IL-23 receptor (IL-23R), RORC (RORγt) and CD161 (KLRB1), whereas RORA (RORα) and STAT3 expression were equivalent. Similar to CD4+ memory T cells, IL-23 responsiveness is confined to the CD161+ subset in CD8+CD45RO+ memory T cells, suggesting a similar CD161+ precursor as has been reported for CD4+ Th17 cells. We observed a very strong positive correlation between IL-23 responsiveness and the fraction of CD161+, CD8+CD45RO+ memory T cells (r = 0.80, p<0.001). Moreover, the fraction of CD161+, CD8+CD45RO+ memory T cells gradually decreases with aging (r = −0.34, p = 0.05). Our data define the inter-individual differences in IL-23 responsiveness in peripheral blood lymphocytes from the general population. Variable expression of CD161, IL-23R and RORC affects IL-23 responsiveness and contributes to the inter-individual susceptibility to IL-23-mediated defenses and inflammatory processes.

## Introduction

Interleukin-23 (IL-23) is a member of the IL-12 cytokine family and is comprised of p19 (IL23A) and p40 (IL12B) subunits. By binding to a heterodimeric receptor complex composed of IL-12Rβ1 and IL-23R (IL-23 receptor), IL-23 activates the Janus kinase 2 (JAK2) and tyrosine kinase 2 (TYK2), which in turn leads to the recruitment and phosphorylation of signal transducer and activator of transcription signaling molecules (STATs), in particular STAT3, but also STAT1, STAT4 and STAT5 depending on the cell type [1].

Genome-wide association studies have demonstrated highly significant associations between polymorphisms in IL-23 pathway genes, notably IL-23R, to a number of chronic inflammatory disorders, including inflammatory bowel disease (IBD) [2], psoriasis [3], and ankylosing spondylitis [4]. For instance, multiple single nucleotide polymorphisms (SNPs) in the IL-23R gene demonstrate significant, independent evidence for association with Crohn’s disease [2]. Interestingly, three other IL-23 pathway genes, including IL12B, STAT3 and JAK2, have also been definitively associated with Crohn’s disease, highlighting a central role for IL-23 signaling in disease pathogenesis [5].

Genetic advances have paralleled findings from *in vivo* models that have established an essential role for IL-23 in mediating end-organ inflammation involving the intestine [6]–[9], joints [10], and central nervous system [11]. Increased expression of the IL-23A subunit results in multi-organ inflammation, including intestinal inflammation [12]. Conversely, an intact IL-23 pathway is required for maximal disease expression in a variety of murine models, including both T cell and non-T cell mediated models of intestinal inflammation [6]–[9]. A pro-inflammatory role for IL-23 responsive CD4+ Th17 cells has been established for a variety of inflammatory disorders. The regulated expression of IL-23R represents a key differentiation step, regulated in part by the transcription factors RORγt [13], RORα [14] and STAT3 [15]. IL23R is expressed on multiple cell populations, including CD4+ and CD8+ memory T cells [1], and NKT (natural killer T) cells [16], and stimulation with IL-23 contributes to various functions within these immune cell subsets, including expansion, stabilization and cytokine secretion.

These genetic and molecular studies have unequivocally demonstrated that IL-23 pathway plays a critical role in the pathogenesis of multiple chronic inflammatory disorders. This would imply that differences in IL-23-mediated signaling activity may underlie variable susceptibility to these disorders. However, inter-individual variability in IL-23-induced signal transduction in circulating human lymphocytes has not been well-defined. In this study, we observed marked, reproducible inter-individual differences in IL-23-mediated STAT3 phosphorylation in human peripheral blood CD8+CD45RO+ memory T and CD3+CD56+ NKT cells, with decreased IL-23 responsiveness observed with increased age and with decreased CD161, IL23R and RORC expression. In addition, as has been reported for CD4+ memory T cells, the IL-23 response is confined to the CD161+ subset of CD8+CD45RO+ memory T cells.

## Results

### Significant, Reproducible Inter-individual Variability in IL-23-mediated STAT Phosphorylation in CD8+CD45RO+ Memory T and NKT Cells from Healthy Controls

**Figure 1:**
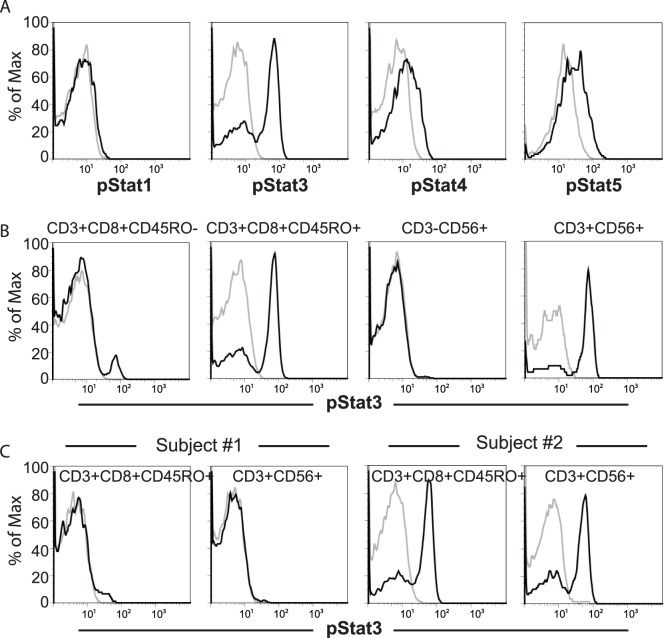
IL-23 stimulation of peripheral blood lymphocytes activates STAT proteins with inter-individual variability in IL-23 responsiveness. Peripheral whole blood was stimulated with 100ng/ml IL-23 for 15 minutes and IL-23-mediated pSTAT induction was assessed by phospho-flow assays for STAT1, 3, 4 and 5. (A) IL-23-mediated pSTAT induction in CD8+CD45RO+ memory T cells. Gray line and black lines represent pSTAT in un-stimulated and IL-23 stimulated samples, respectively. (B) IL-23-mediated pSTAT3 induction in CD8+ naïve (CD45RO-) and memory (CD45RO+) T cells, NK cells (CD3−CD56+), and NKT (CD3+CD56+) cells. (C) Representative individuals showing low (subject #1) or high (subject #2) IL-23 responsiveness.

To better define IL-23-mediated signaling transduction in primary human immune cells, we assessed intracellular STAT phosphorylation (pSTATs) in whole blood stimulated with IL-23 for 15 minutes, the time point we found to result in optimal IL-23-induced STAT activation (Figure S1). Consistent with prior reports in cell lines [1], IL-23 stimulation induced robust STAT3 phosphorylation, as well as STAT4 and STAT5 phosphorylation in CD8+CD45RO+ memory T cells (Figure 1A) and CD3+CD56+ NKT cells (pSTAT3 shown in Figure 1B, data for pSTAT4-5 not shown). However, minimal or no activation of STAT1 was observed (Figure 1A). Most CD8+CD45RO- naïve T cells and CD3−CD56+ NK cells did not demonstrate pSTAT3 (Figure 1B) or pSTAT1/4/5 (data not shown) induction in response to IL-23 stimulation (Figure 1B). As pSTAT3 demonstrated the most robust activation to IL-23, subsequent analyses were focused on pSTAT3. Significant inter-individual differences were observed in IL-23-mediated STAT3 activation (Figure 1C), with some individuals (e.g. Subject #1 in Figure 1C) demonstrating minimal to no induction of pSTAT3, while others (e.g., Subject #2 in Figure 1C) demonstrated strong induction of pSTAT3.

**Figure 2:**
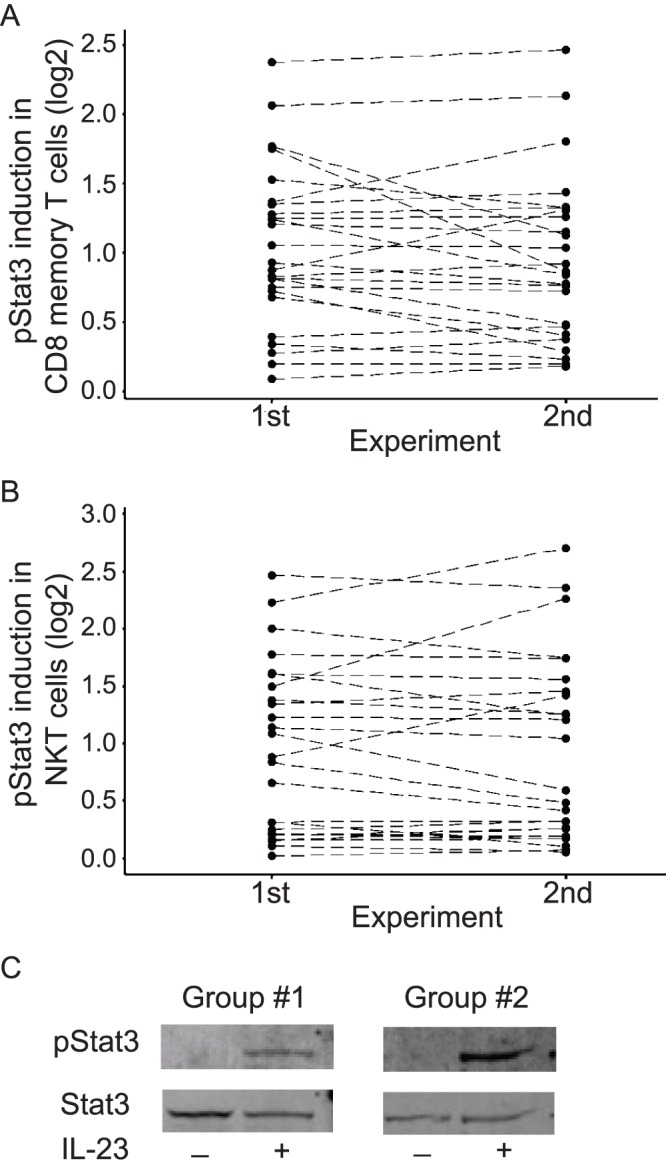
Reproducibility of IL-23 responsiveness as assessed by pSTAT3 induction. Peripheral whole blood was stimulated with 100 ng/ml IL-23 for 15 minutes and pSTAT3 induction was assessed by phospho-flow assay. Repeated measures of IL-23-mediated pSTAT3 induction were performed for 25 randomly selected individuals, with the first and the second experiments separated by at least a three-week interval. IL-23 responsiveness was calculated as the log2 ratio of geometric mean fluorescence intensity (GMFI) of pSTAT3 in stimulated vs. unstimulated samples for (A) CD8+CD45RO+ memory T cells, and (B) CD3+CD56+ NKT cells. (C) IL-23-mediated pSTAT3 induction assessed by Western blot analysis in FACS sorted CD8+CD45RO+ memory T cells from individuals showing low (Group #1) or high (Group #2) IL-23-mediated pSTAT3 induction in phospho-flow assays.

To assess intra-individual reproducibility of IL-23-mediated STAT3 activation, we repeated the whole blood phospho-flow assays in 25 randomly selected individuals, allowing for at least a three-week interval between replicate studies (Figure 2). The degree of IL-23-mediated pSTAT3 induction was quantitatively measured as the log 2 ratio of the geometric mean fluorescent intensity (GMFI) values of pSTAT3 in IL-23 stimulated cells over unstimulated cells. In CD8+CD45RO+ memory T cells (Figure 2A) and CD3+CD56+ NKT (Figure 2B) cells, intra-individual reproducibility was excellent, such that no significant difference in IL-23 responsiveness was detected between the first and second experiments across the individuals examined (p = 0.18 and 0.85, respectively). In contrast, highly significant inter-individual differences were observed (p<0.001) in both cell subsets. In addition, we carried out the phospho-flow assays under a number of different conditions by varying IL-23 doses or stimulation time (Figure S1) and demonstrated that the observed inter-individual differences in IL-23 responsiveness are not dependent on IL-23 doses or stimulation time. To confirm the trends observed by whole blood phospho-flow assays, we performed western blot analysis of sorted CD8+CD45RO+ memory cells. In individuals demonstrating low (Group #1) and high (Group #2) levels of IL-23-mediated pSTAT3 induction on phospho-flow assays, we observed correlative IL-23-mediated induction of pSTAT3 by western blot analysis (Figure 2C). Taken together, these results demonstrate that peripheral CD8+CD45RO+ memory T and CD3+CD56+ NKT cells from the general population have reproducible, significant inter-individual variability in IL-23-induced STAT3 activation.

### Age Contributes to Variation in IL-23 Responsiveness in CD8+CD45RO+ Memory T Cells

**Figure 3:**
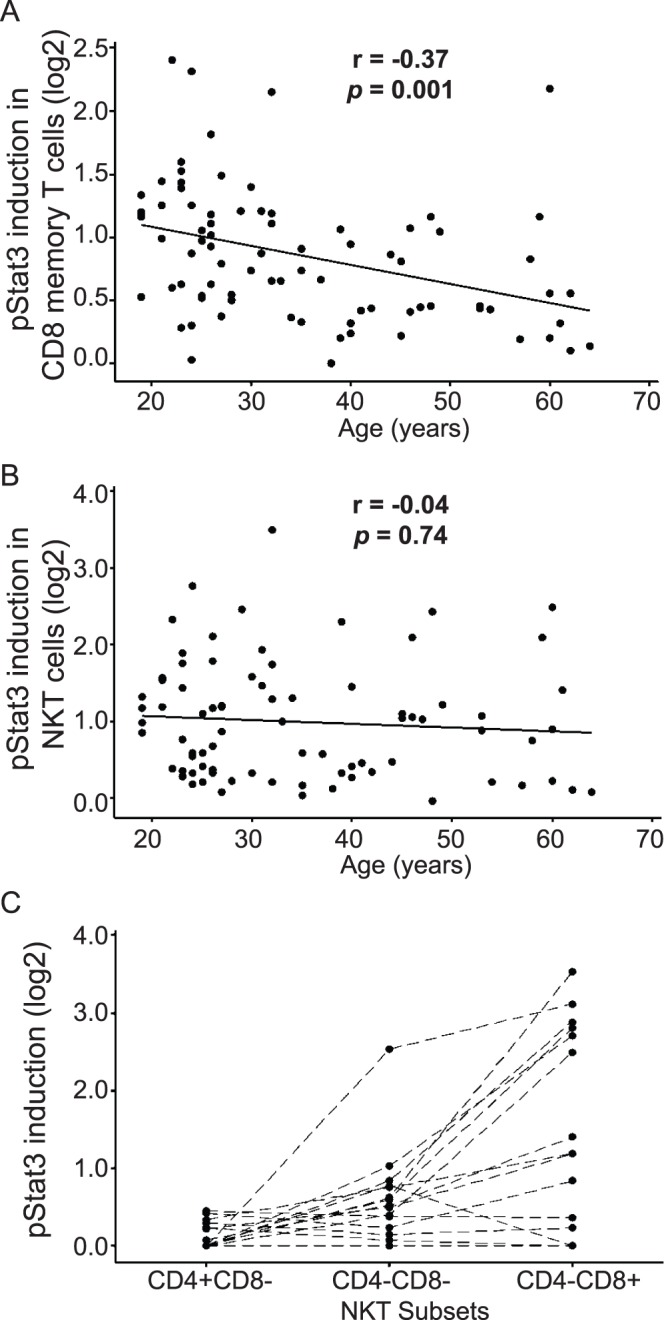
Age-related correlation and subset analysis for IL-23-mediated pSTAT3 induction in CD8+CD45RO+ memory T cells and CD3+CD56+ NKT cells. Peripheral whole blood was stimulated with 100 ng/ml IL-23 for 15 minutes and pSTAT3 induction was assessed by phospho-flow assay. IL-23 responsiveness was calculated as the log2 ratio of GMFI of pSTAT3 in stimulated vs. unstimulated samples. Age is significantly associated with IL-23 responsiveness in (A) CD8+CD45RO+ memory T cells (n = 82, r = −0.37, p = 0.001), but not in (B) CD3+CD56+ NKT cells (n = 82, p = 0.50). (C) IL-23 responsive NKT cells are confined to the CD4−CD8+ and CD4−CD8− subsets (n = 20).

We next considered factors that might contribute to the significant inter-individual variability in IL-23 responsiveness observed in primary human cells. As it is known that gender and aging may contribute to inter-individual variability in human immune responses [17]–[20], we evaluated gender and age as potential factors contributing to inter-individual variability in IL-23 responsiveness in 82 randomly selected healthy individuals. There was no significant difference in IL-23 responsiveness between males (n = 24) and females (n = 58) (t-test, p = 0.18 and 0.38 in CD8+CD45RO+ memory T cells and CD3+CD56+ NKT cells, respectively, data not shown). However, in CD8+CD45RO+ memory T cells, we observed a modest but significant correlation of decreased IL-23 responsiveness with increasing age (Pearson’s correlation coefficient r = −0.37, p = 0.001, Figure 3A).

We find that IL-23-mediated activation of STAT3 in CD3+CD56+ NKT cells is strongly correlated with that in CD8+CD45RO+ memory T cells (r = 0.70, p<0.001, Figure S2). However, in contrast to CD8+CD45RO+ memory T cells, the IL-23 responsiveness in CD3+CD56+ NKT cells did not show significant correlation with age (p = 0.50, Figure 3B). Because it is known that CD3+CD56+ NKT cells also contain a subset of CD8+ cells, we further investigated the IL-23 responsiveness in NKT cell subsets. Within CD3+CD56+ NKT cells, CD4+CD8−, CD4−CD8+ and CD4−CD8− subsets are observed. Evaluation of a separate cohort of 20 individuals revealed that the IL-23 responsive NKT subsets are confined to the CD4−CD8+ and CD4−CD8− NKT subpopulations (Figure 3C). Therefore, the observed correlation in IL-23 responsiveness between CD3+CD56+ NKT cells and CD8+CD45RO+ memory T cells is partially attributable to a common subset of cells (i.e., CD3+CD8+CD56+CD45RO+). Among CD3+CD56+ NKT subsets, on average, 34.4% (standard deviation, SD: 16.4%, range: 13.5–71.4%) are CD8+CD45RO+ (Table S1 and Figure S3). Conversely, in the CD8+CD45RO+ memory T cells, on average 6.47% (SD: 4.62%, range: 1.21–16.31%) of these cells are CD56+ (Table S1 and Figure S3), consistent with prior reports [21], [22].

### Expression of KLRB1 (CD161), IL-23R and RORC (RORγt) are Significantly Correlated with IL-23 Responsiveness in CD8+CD45RO+ Memory T Cells

To define mechanisms that might be contributing to the differential IL-23-induced STAT3 activation between individuals, we examined mRNA expression differences in CD8+CD45RO+ memory T cells between IL-23 responsive and non-responsive individuals. We performed a microarray analysis of unstimulated and IL-23 stimulated CD8+CD45RO+ memory T cells from two individuals demonstrating robust IL-23 responsiveness, and two individuals demonstrating low IL-23 responsiveness. Four genes showed genome-wide significant (p-value <0.05 after Bonferroni correction) differential expression between IL-23-responsive and -non-responsive individuals (Table 1), including two transcription factors, CEBPD (CCAAT/enhancer binding protein, delta) and ZBTB16 (zinc finger and BTB domain containing 16), and two transmembrane proteins, MPZL3 (myelin protein zero-like 3) and KLRB1 (killer cell lectin-like receptor subfamily B, member 1, CD161).

**Table 1:** Selected results of differential gene expression analysis in CD8+CD45RO+ memory T cells from IL-23-responsive *vs.* -non-responsive individuals.

Gene	P-value^1^	Fold change^2^
CEBPD, CCAAT/enhancer binding protein (C/EBP), delta	8.64E-07	4.61
ZBTB16, zinc finger and BTB domain containing 16	1.58E-06	6.16
MPZL3, myelin protein zero-like 3	2.46E-06	1.51
KLRB1 (CD161), killer cell lectin-like receptor subfamily B, member 1	3.87E-06	3.83
IL-23R, interleukin 23 receptor	3.41E-03	2.94
RORC (RORγt), RAR-related orphan receptor C	6.27E-04	4.09
RORA, RAR-related orphan receptor A	1.72E-03	1.55
TBX21 (T-bet), T-box 21	9.30E-03	−1.65
IFNG, interferon, gamma	0.41	–
STAT3, signal transducer and activator of transcription 3	0.19	–
JAK2, Janus kinase 2 (a protein tyrosine kinase)	0.34	–
TYK2, tyrosine kinase 2	0.24	–
IL12RB1. interleukin 12 receptor, beta 1	0.55	–

1 Genome-wide significance threshold (by Bonferroni correction): p = 0.05/11740 = 4.26×10^−6^.

2 Fold change is calculated as (mean expression level in IL-23-responsive subjects/mean expression level in IL-23-non-responsive subjects).

**Figure 4:**
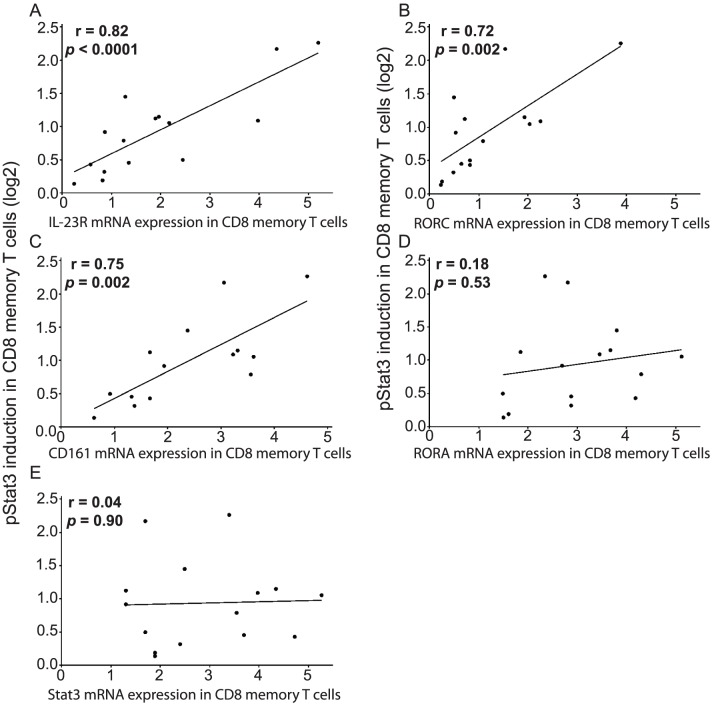
IL-23 responsiveness in CD8+CD45RO+ memory T cells is correlated with IL-23R, RORC and CD161 mRNA expression. Quantitative RT-PCR assays were performed in FACS-sorted CD8+CD45RO+ memory T cells from 15 individuals selected for having a range of IL-23 responsiveness, which were measured by whole blood phospho-flow assays for IL-23-mediated pSTAT3 induction (log2). Correlations were assessed between IL-23 responsiveness and (A) IL-23R, (B) RORC, (C) CD161, (D) RORA, and (E) STAT3 mRNA expression.

We also examined the microarray data for differential expression of select genes of known relevance to the IL-23 pathway (Table 1 and Table S2). Compared to IL-23-non-responsive individuals, we observed increased mRNA expressions of IL-23R, RORC (RORγt, RAR-related orphan receptor C), and RORA (RAR-related orphan receptor A) as well as reduced expression of TBX21 (T-box 21, also known as T-bet) in IL-23-responsive subjects (Table 1). No differential expression was detected for other members of the IL-23 pathway (e.g., IL12RB1, JAK2, TYK2, STAT3, CCR6 or IL17), nor for other Tc1-associated genes (e.g., IFNG, perforin, or granzyme B), between the IL-23-non-responsive and the responsive subjects (Table 1 and Table S2). To further evaluate the correlation between expression levels of these IL-23 pathway genes and IL-23 responsiveness, we performed real-time RT-PCR assays for selected genes in CD8+CD45RO+ memory T cells sorted from 15 individuals that had been selected for having a range of different IL-23 responsiveness. Significant positive correlations were observed between IL-23 responsiveness and mRNA levels for IL-23R (Figure 4A, r = 0.82, p<0.001), RORC (Figure 4B, r = 0.72, p = 0.002) and CD161 (Fig. 4C, r = 0.75, p = 0.002). In contrast, we did not observe a significant correlation in IL-23 response with RORA or STAT3 mRNA expressions (Figure 4D–4E). Therefore, CD8+CD45RO+ memory T cells with increasing IL-23 responsiveness demonstrate increasing levels of CD161, IL-23R, and RORC mRNA expression.

### The CD161+ Subset in CD8+CD45RO+ Memory T Cells Represents the IL-23 Responsive Cell Subset and Decreases in Fraction with Increasing Age

**Figure 5:**
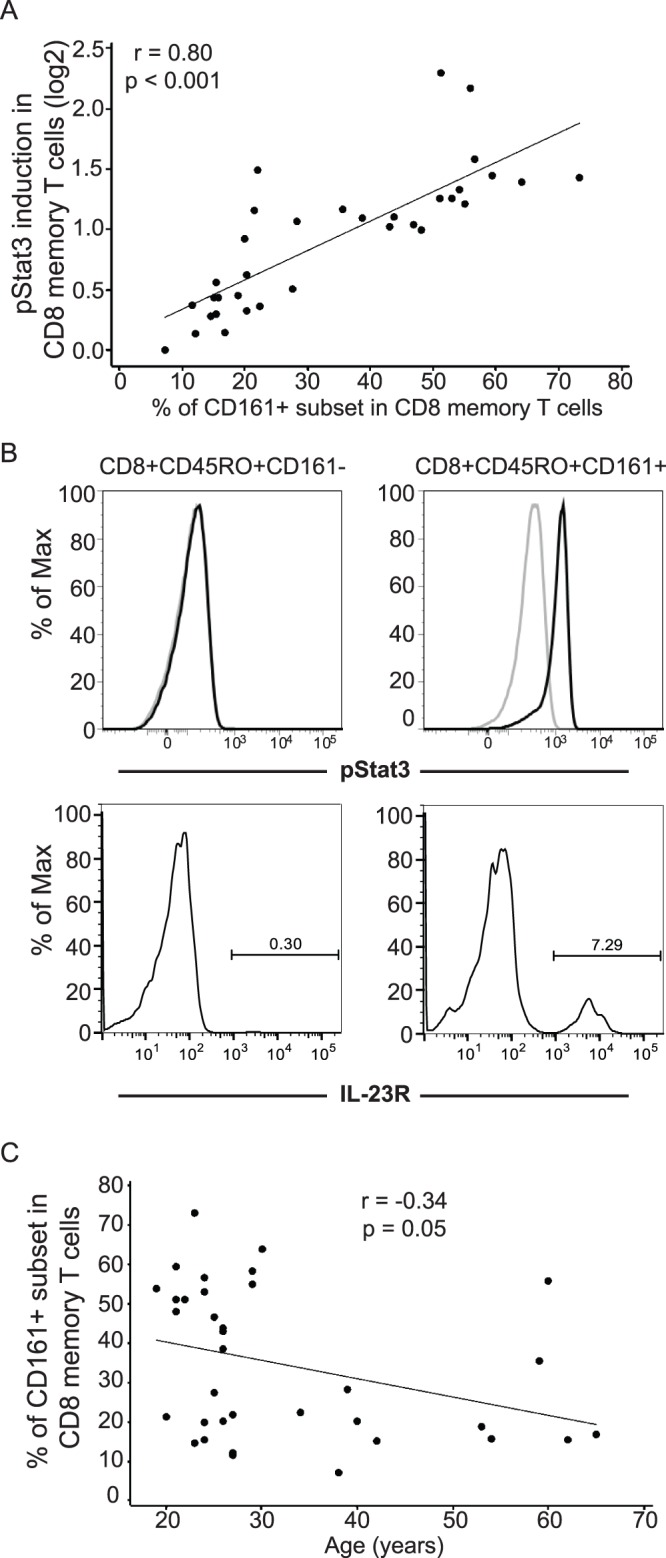
The fraction of CD161+ subset is correlated with IL-23 responsiveness in CD8+CD45RO+ memory T cells and with age. (A) The fraction of the CD161+ subset in CD8+CD45RO+ memory T cells is positively correlated (n = 35, r = 0.80; p<0.001) with IL-23 responsiveness, measured by whole blood phospho-flow for IL-23-mediated pSTAT3 induction (log2). (B) Upper panel: FACS-sorted CD3+CD8+CD45RO+ CD161+ or CD161− T cells were stimulated with or without 100 ng/ml IL-23 for 15 minutes and pSTAT3 induction was assessed by phospho-flow. Representative flow cytometry plots are shown from one of the three individuals assessed. IL-23 responsive CD8+CD45RO+ T cells are confined to the CD161+ fraction. Lower panel: the percent IL-23R-expressing cells in CD8+CD161− or CD8+CD161+ cells are shown. Representative flow cytometry plots are shown from one of the two individuals assessed. (C) The fraction of the CD161+ subset in CD8+CD45RO+ memory T cells gradually declines with age (n = 35, r = −0.34, p = 0.05).

The observed positive correlations of CD161, IL-23R and RORC expression with IL-23 responsiveness in CD8+CD45RO+ memory T cells correspond with reports [23]–[26] which indicated that CD161 expression defines a distinct subset in CD4+/CD8+ T cells expressing high levels of IL-23R and RORC and containing virtually all populations of IL-17-secreting T cells. We therefore asked to what extent inter-individual variability in IL-23 responsiveness is correlated with the fraction of the CD161+ subset in CD8+CD45RO+ memory T cells. In a randomly selected 35 individuals, we observed a very strong positive correlation between IL-23 responsiveness and the fraction of the CD161+ subset in CD8+CD45RO+ memory T cells (Figure 5A, r = 0.80, P value <0.001). To further define the unique responsiveness of CD161+CD8+CD45RO+ T cells to IL-23 stimulation, we sorted CD3+CD8+CD45RO+CD161− and CD3+CD8+CD45RO+CD161+ cell subsets and examined STAT3 activation to IL-23 stimulation. We found that IL-23-mediated STAT3 activation was universally observed in the CD161+ fraction, whereas no IL-23 response was seen in the CD161− fraction of CD8+CD45RO+ T cells (Figure 5B). Consistently, CD161+CD8+CD45RO+ T cells exhibit a higher percentage of IL-23R-expressing cells compared to the CD161−CD8+CD45RO T cells (Figure 5B).

Given the observed correlation of decreasing IL-23 responsiveness in CD8+CD45RO+ memory T cells with increasing age, we next assessed whether and to what extent the fraction of CD161+ subset in CD8+CD45RO+ memory T cells decreases with age. Similar to the age-dependent effects observed in IL-23 responsiveness, we observe a modest but significant negative correlation (Figure 5C) between the percentage of CD161+ cells within CD8+CD45RO memory T cells and age (n = 35, r = −0.34, p = 0.05). Therefore, the decreasing IL-23 responsiveness in CD8+CD45RO+ T cells with increasing age is at least partially due to an age-dependent decline in the fraction of CD161+ subset within the CD8+CD45RO+ memory T cells.

## Discussion

In this study, we demonstrate significant, reproducible inter-individual variation in IL-23-mediated signaling transduction (STAT3 phosphorylation) in both primary human CD8+CD45RO+ memory T cells and CD3+CD56+ NKT cells. The inter-individual variability in IL-23 responsiveness in CD8+CD45RO+ memory T cells is at least partially due to an age-dependent decline in the fraction of CD161+ subset within the CD8+CD45RO+ memory T cells. Importantly, we now also show that similar to CD4+ memory T cells [23], [26], IL-23 responsiveness is uniquely confined to the CD161−expressing subpopulation in CD8+CD45RO+ memory T cells. The observed strong positive correlations of IL-23 responsiveness with CD161, IL-23R and RORC expression levels in CD8+CD45RO+ memory T cells imply that elevated CD161 expression is associated with increased levels of IL-23R and RORC expression, expression of which is enriched in Th17 cells. On the other hand, IL-23 responsive subjects showed reduced expression of TBX21/T-bet, which is a master regulator of Tc1 differentiation and prevents Tc17 or Tc2 differentiation [27]. The IL-23 responsivity in the CD161+ population is consistent with several recent reports [23], [28] suggesting CD161 as a hallmark of cells polarized toward Th17 lineage in CD4+ and CD8+ T cells. Interestingly, age-dependent decreases of CD161 expression have been reported in CD4+ memory T cells [25], [29], as well as in CD8+ memory T cells [30]. On the other hand, enrichment of CD161+ T cells has been observed in tissues samples of several chronic inflammatory diseases [23]–[26], such as Crohn’s disease and multiple sclerosis. A variety of age-dependent alternations in the immune system have been associated with the increased susceptibility to infections and neoplasia with advancing age [31], [32]. Given the central role of the IL-23 pathway in IBD, it may be speculated that a decrease in CD161 expression/IL-23 responsiveness with increasing age may partially explain the decreased disease incidence observed after the peak age of onset in childhood and early adulthood. On the other hand, given the importance of the IL-23 pathway in mediating antimicrobial defenses, it may also contribute to the increased risk of infection with increasing age. However, it is not clear what causes such change in CD161 expression and/or IL-23 responsiveness. The ligand for CD161 has been identified to be lectin-like transcript 1 (LLT1) [33], [34], and LLT1 engagement of CD161 on T cells in the presence of T cell receptor stimulation leads to enhanced proliferation, and increased IL-17 and IFN-γ production by the T cells [33], [35]. Possible additional roles include promoting transendothelial migration [36]. Whether the concordance between CD161 expression levels and IL-23 responsiveness observed in this study merely reflects CD161’s role as a marker for lineage-specificity, or results from an essential functional role of CD161-dependent signaling has yet to be determined.

Not all transcription factors which contribute to IL-23R expression were differentially expressed between IL-23-responsive and –non-responsive subjects in CD8+CD45RO+ memory T cells. While RORC, RORA and STAT3 genes have been implicated in regulating IL-23R expression [14], [15], we only observed robust association between RORC mRNA expression and IL-23 responsiveness. In addition, we observed increased expression of various transcription factors that have not been previously implicated in regulating IL-23 signal transduction. The increased expression of CEBPD (CCAAT/enhancer binding protein, delta) in IL-23-responsive individuals is potentially of interest given prior reports of its role downstream of both IL-1β and IL-6 signaling [37]; both of these cytokines have been implicated in CD4+ Th17 differentiation [38], [39]. Finally, ZBTB16 is also increased in IL-23-responsive compared to non-responsive individuals. ZBTB16 is a zinc-finger protein transcription factor family member and has been implicated in the effector program of NKT cells [40]. Taken together, the increased expression of CD161, RORC, IL-23R, and ZBTB16 highlights shared transcriptional regulation between NKT and IL-23 responsive CD8+CD45RO+ memory T cells.

In addition to increased CD161, another differentially expressed transcript common to the present microarray analysis and a previously reported CD4+ Th17 comparative microarray analysis [23] is that of increased expression of GALNT10 (UDP-N-acetyl-alpha-D-galactosamine:polypeptide N-acetylgalactosaminyltransferase 10) (Table S2). In the prior study [23], GALNT10 was increased in Th17 compared to Th1 clones. GALNT10 initiates the synthesis of mucin-type oligosaccharides by transferring GalNAc [41].

It was previously reported that IL-23 induces STAT1, STAT3, STAT4 and STAT5 phosphorylation in human T cell lines [1]. Consistent with these findings, we also observed robust STAT3, STAT4 and STAT5 phosphorylation in response to IL-23 stimulation in primary human CD8+CD45RO memory T cells and CD3+CD56+ NKT cells. However, we detected minimal to no STAT1 activation. Interestingly, two studies from the same group have shown that IL-23 induces phosphorylation of STAT3 and STAT4 but not STAT1 or STAT5 in human primary CD3+CD56+ T cells [42], and phosphorylation of STAT1, STAT3 and STAT4, but not STAT5, in T cell blasts transduced with IL-23R [43]. The reasons for the observed discrepancy in IL-23-induced STAT1 and STAT5 phosphorylation are unclear at this time, but are possibly related to differences in cell types and experimental conditions (e.g., IL-23 doses).

In CD3+CD56+ NKT cells, IL-23-induced STAT3 phosphorylation was observed in CD4−CD8+ and CD4−CD8− subsets, but not in the CD4+CD8− subset. Furthermore, a fraction of CD4−CD8+ and CD4−CD8− NKT subsets also express CD161 (Table S1), which is consistent with previous reports [21], [44]. CD4−CD8+ and CD4−CD8− NKT subsets have been reported to have increased cytotoxicity and increased secretion of select cytokines relative to the CD4+CD8− subset, with CD4−CD8− NKT cells having particularly enhanced IL-17 secretion [45]. Moreover, human NKT cells require various factors in order to optimally produce IL-17, include stimulation by IL-23 [46].

In summary, we defined significant, reproducible inter-individual variation in IL-23-mediated signaling transduction in primary human CD8+CD45RO+ memory T cells and CD3+CD56+ NKT cells from healthy individuals. IL-23 responsiveness in CD8+CD45RO+ memory T cells is confined to the CD161+ subset, whose fraction within the CD8+CD45RO+ memory T cells gradually declines with aging. Variable expression of CD161, IL-23R and RORC affects IL-23 responsiveness and likely contributes to the inter-individual susceptibility to IL-23-mediated defenses and inflammatory processes. The IL-23/Th17 pathway contributes to multiple autoimmune and inflammatory diseases. Furthermore, the identification that IL-23R disease protective polymorphisms result in a loss-of-function resulting in decreased IL-23R signaling and decreased Th17 and Tc17 cells [47]–[49], highlights the importance of the relationship between inter-individual variation in this pathway to inflammatory disease susceptibility. The current studies provide insight into additional contributions to the important inter-individual variation in the IL-23 pathway responses.

## Materials and Methods

### Human Subjects

This study has been approved by Yale University Institutional Review Boards. A written informed consent has been obtained from each individual healthy donor, and the consent procedure has also been approved by Yale University Institutional Boards. To minimize ethnic difference, all the subjects are limited to Caucasians of European ancestry. The study included 82 healthy, unrelated donors, each with no personal or family history of IBD, and no personal history of psoriasis, systemic lupus erythematosus, rheumatoid arthritis, multiple sclerosis or HIV.

### Antibodies

Alexa647-conjugated phospho-specific monoclonal antibodies against pSTAT1 (pY701, clone 4a), pSTAT3 (pY705, clone 4), pSTAT4 (pY693, clone 38), pSTAT5 (pY694, clone 47) and surface marker antibodies to human CD3-PerCPCy5.5 (SK7), CD3-PECy7 (SK7), CD4-Pacific Blue (RPA-T4), CD4-V500 (RPA-T4), CD8-FITC (RPA-T8), CD8-APC (RPA-T8), CD8-PerCP (RPA-T8), CD45RO-PE (UCHL1), CD161-PE (DX12), and CD161-FITC (DX12) were obtained from BD Biosciences (San Jose, CA). FITC-conjugated anti-human CD56 (clone C5.9) was purchased from Sigma (St Louis, MO). Anti-human CD45RO-Pacific Blue (UCHL1) was obtained from BioLegend (San Diego, CA). IL-23R-APC (clone 218213) was obtained from R&D Systems (Minneapolis, MN).

### Whole Blood Phospho-Flow Assay

Intracellular phospho-epitopes were measured using whole blood phospho-flow. Briefly, whole blood samples were left untreated or stimulated with recombinant human IL-23 (R&D Systems, Minneapolis, MN). After the stimulation, cells were immediately fixed and washed, and then permeablized by using BD Phosflow buffers (BD Biosciences). After permeabilization, cells were stained with corresponding surface and intracellular phospho-antibodies and analyzed using FACSCalibur or LSRII flow cytometer (BD Biosciences). FlowJo (Tree Star, Ashland, OR) was used for data analysis. CD8 naïve and memory T cells were gated as CD3+CD8+CD45RO- and CD3+CD8+CD45RO+, respectively. NK and NKT cells were gated as CD3+CD56− and CD3+CD56+, respectively. For both unstimulated and IL-23 stimulated cell subsets, the GMFI values of pSTATs were computed. IL-23 responsiveness was calculated as the log2 ratio of GMFI of pSTAT3 in stimulated vs. unstimulated samples.

### Fluorescence-activated Cell Sorting and Analysis

Peripheral blood mononuclear cells (PBMC) were isolated by Ficoll (***Ficoll*** **-**Paque PREMIUM, GE healthcare, Piscataway, NJ) density gradient separation. PBMC were stained with CD3-PECy7, CD8−APC, and CD45RO-Pacific Blue, and CD8 memory T cells were then separated by cell sorting with a FACSAria cell-sorting system. In some cases, CD161 staining was added to further sort CD161+ and CD161- memory CD8+ T cells. Sorted cells were subjects to RNA or protein isolation for real-time RT-PCR or Western blotting, respectively or phospho-flow.

### Western Blotting

FACS Sorted CD8+CD45RO+ memory T cells were either left untreated or treated with 100 ng/ml IL-23 for 15 min at 37°C. Because of low amount of total protein isolated from each subject due to limited number of cells, we pooled four protein samples into groups of low or high IL-23 responders (two samples in each group) based on the phospho-flow data. Western blotting was carried out using protocols established in our previous studies [50].

### Gene Expression Profiling

Sorted CD8+CD45RO+ memory T cells were either left untreated in RPMI-1640 complete medium or treated with IL-23 at 100 ng/ml for 6 h at 37°C. RNA was extracted by RNeasy Mini kit (Qiagen, Valencia, CA) and tested on GeneChip ***Human Exon*** 1.0 ST Arrays (Affymetrix, Santa Clara, CA) by the Microarray Core Facility at Yale Keck Biotechnology Resource Laboratory. The gene-level expression values were estimated by using IterPLIER algorithm implemented in the Affymetrix Expression Console software. Since results from genes with low expression values close to the noise level may not be reliable, only genes with minimum gene expression signals of 20 were called “present” and subjected to further analyses. This threshold was estimated based on the median expression signals of negative control probe sets. After excluding “absent” gene probe sets and Affymetrix control probe sets (spike, negative, and positive controls), a total of 11740 gene probe sets (Table S2) were tested for differential expression using Partek Genomics Suite (Partek Incorporated, St. Louis, MO). Genome-wide significance threshold was determined using a Bonferroni correction (p = 0.05/11740 = 4.26×10^−6^). All microarray data is MIAME compliant and the raw data has been deposited in GEO database. The GEO accession number is GSE42051.

### Real-Time RT-PCR

RNA was isolated from sorted CD8+CD45RO+ memory T cells by RNeasy Mini Kit (Qiagen, Valencia, CA), and was reverse transcribed to cDNA with TaqMan Reverse Transcription Reagents (Applied Biosystems, Foster City, CA). Quantitative RT-PCR was performed with TaqMan gene expression assays (Applied Biosystems) on an ABI PRISM 7900HT Sequence Detection System. The following TaqMan gene expression assays were used (Applied Biosystems assay identification numbers in parentheses): IL-23R (Hs01001361_ml), RORC (Hs01076112_ml), RORA (Hs00536545_ml), CD161 (Hs00174469_m1), and STAT3 (Hs00374280_ml). For each sample, mRNA expression was normalized to beta-actin.

### Statistical Analyses

Two-way ANOVA was employed to test the significance of intra-individual reproducibility and inter-individual difference of IL-23 responsiveness in CD8+CD45RO+ memory T cells and CD3+CD56+ NKT cells. Two-sample t-test was used to test the difference of IL-23 responsiveness between males and females. Correlation analyses were carried out between IL-23 responsiveness and various factors, such as age, mRNA expression levels of several genes, and percentage of CD161+ subset in CD8+CD45RO+ memory T cells using Minitab v15 (Minitab Inc, State College PA).

## Supporting Information

Figure S1 Inter-individual variability in IL-23 responsiveness in CD8+CD45RO+ memory T cells and CD3+CD56+ NKT cells is independent of IL-23 doses and stimulation time. A-B. Peripheral blood samples from two healthy individuals were stimulated with various concentrations of IL-23 for 15 minutes. IL-23-induced STAT3 phosphorylation was assessed by phosphor-flow assay. C-D. Peripheral blood samples from two healthy individuals were stimulated with Il-23 (100 ng/ml) for different time periods. IL-23-induced STAT3 phosphorylation was assessed by phosphor-flow assay.
(EPS)

Figure S2 Correlation of IL-23 responses between CD8+CD45RO+ memory T cells and CD3+CD56+ NKT cells. Peripheral whole blood samples were stimulated with 100 ng/ml IL-23 for 15 minutes and pSTAT3 induction was assessed by phospho-flow assay. IL-23 responsiveness was calculated as the log2 ratio of GMFI of pSTAT3 in stimulated vs. unstimulated samples, in CD8+CD45RO+ memory T cells and CD3+CD56+ NKT cells, respectively.
(EPS)

Figure S3 Sample plot of subset gating in CD8+CD45RO+ memory T cells and CD3+CD56+ NKT cells. Peripheral whole blood samples were stimulated with 100 ng/ml IL-23 for 15 minutes and pSTAT3 induction was assessed by phospho-flow assay. Percentages are shown from the parent population, which is calculated by dividing the number of cells in the subpopulation by the number of cells in its direct ancestor population.
(EPS)

Table S1 Overlap between CD8+CD45RO+ memory T cells, CD3+CD56+ NKT cells, and CD3+CD161+ cells in peripheral blood.
(PDF)

Table S2 Genes differentially expressed between IL-23-responsive and non-responsive subjects.
(XLSX)
