# Supplementary material for: Age and CD161 Expression Contribute to Inter-Individual Variation in Interleukin-23 Response in CD8+ Memory Human T Cells
Source: PLoS One. 2013 Mar 1;8(3):e57746. doi: 10.1371/journal.pone.0057746 (PMC3585933; doi:10.1371/journal.pone.0057746)
Supplement: Table S1 — Overlap between CD8+CD45RO+ memory T cells, CD3+CD56+ NKT cells, and CD3+CD161+ cells in peripheral blood. (PDF) [file pone.0057746.s004.pdf]

Table S1. Overlap between CD8+CD45RO+ memory T cells, CD3+CD56+ NKT cells, and CD3+CD161+ cells in peripheral blood

	**Mean**	**SD**	**Range**
# of healthy donors	15		
Age (years)	31.4	12.21	19-60
CD3+CD8+CD45RO+ memory T			
CD56+	6.47 %	4.62 %	1.21 – 16.31 %
CD161+	33.70 %	18.06 %	11.52 – 55.86 %
CD56+CD161+	5.16 %	5.00 %	0.49 – 15.61 %
CD3+CD56+ NKT cells			
CD8+CD45RO+	34.38 %	16.41 %	13.52 – 71.35 %
CD161+	51.30 %	23.82 %	16.37 – 85.85 %
CD8+CD45RO+CD161+	26.86 %	19.29 %	1.92 – 66.89 %
CD3+CD161+			
CD8+CD45RO+	23.59 %	9.82 %	9.19 – 43.55 %
CD56+	6.56 %	3.60 %	1.37 – 13.68 %
CD8+CD45RO+CD56+	1.83%	1.53 %	0.13 – 4.58 %

Percentages are fractions of each subset within CD8+CD45RO+ memory T cells, CD3+CD56+ NKT cells, or CD3+CD161+ cells. SD, Standard Deviation.
